# Merkel Cell Carcinoma of the Axilla and Adrenal Gland: A Case Report with Imaging and Pathologic Findings

**DOI:** 10.1155/2015/931238

**Published:** 2015-05-17

**Authors:** Soo Heui Baek, Hyun Kyung Jung, WooGyeong Kim, Suk Jung Kim, Hye Jin Baek, Seung Ho Kim, Yedaun Lee, Young Mi Park

**Affiliations:** ^1^Department of Radiology, Haeundae Paik Hospital, Inje University College of Medicine, Busan 612-896, Republic of Korea; ^2^Department of Pathology, Haeundae Paik Hospital, Inje University College of Medicine, Busan 612-896, Republic of Korea; ^3^Department of Radiology, Busan Paik Hospital, Inje University College of Medicine, Busan 614-735, Republic of Korea

## Abstract

Merkel cell carcinoma (MCC) is a rare and aggressive neuroendocrine carcinoma of the skin. MCC is characterized by a high incidence of locoregional recurrence, and distant metastasis, and often requires short-term follow-up after treatment. In this present paper, we describe a rare case of MCC, which presented as a palpable axillary mass and an incidental adrenal mass, and report on the ultrasonography, computed tomography, and ^18^F-fluorodeoxyglucose-positron emission tomography findings. The patient underwent surgery and adjuvant radiation therapy. Seven months after the initial diagnosis, distant metastasis was detected during a follow-up examination.

## 1. Introduction

Merkel cell carcinoma (MCC) is a rare and aggressive neuroendocrine carcinoma of the skin. MCC usually affects elderly patients, with a mean age of 70 years at the time of diagnosis [1]. It is usually detected on sun-exposed areas of the skin such as the head and neck region (47% of incidences), followed by the extremities (40%) and the trunk (8%) [2]. It is characterized by a high frequency of local recurrence, regional nodal metastasis, distant metastasis, and a low survival rate [2]. Agelli et al. reported that the age-adapted incidence rate of this disease has increased 3-fold, with an annual increase of 8% from 1986 to 2001 [3]. These statistics provide compelling reasons for early diagnosis and disease management for patients with MCC. Moreover, as MCC is a rare skin cancer, suitable imaging modalities have not been fully established [4–8]. In the present paper, we describe a unique case of MCC of the axilla and adrenal gland in a 53-year-old woman, report on the imaging findings, and review the relevant literatures regarding this disease.

## 2. Case Presentation

**Figure 1:**
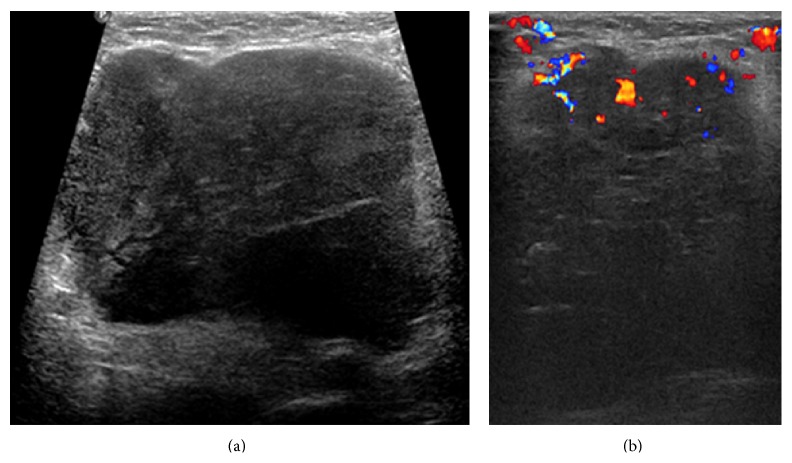
Transverse (a) and color Doppler (b) images of ultrasonography show an irregular mass with an indistinct margin, internal hypoechogenicity, and increased peripheral vascularity.

**Figure 2:**
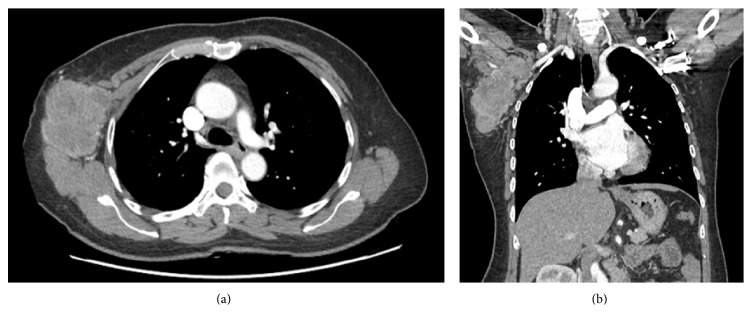
Axial (a) and coronal (b) chest computed tomography images show a 12 cm mass with a lobulated contour and heterogeneous enhancement in the right axilla.

**Figure 3:**
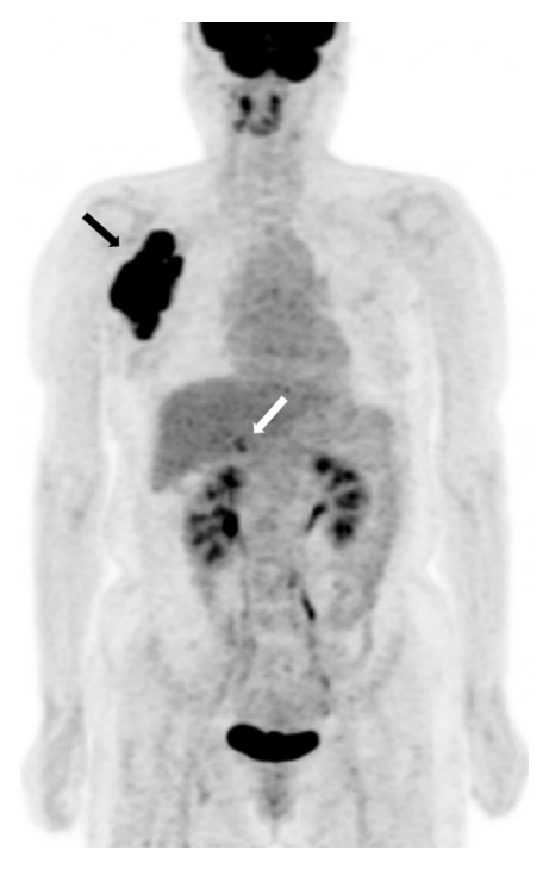
^18^F-FDG-PET scan shows two focal FDG-avid uptakes in the right axilla (SUV max 12.7, black arrow) and right adrenal gland (SUV max 4.7, white arrow). SUV max: maximum standardized uptake value. ^18^F-FDG-PET: ^18^F-fluorodeoxyglucose-positron emission tomography.

**Figure 4:**
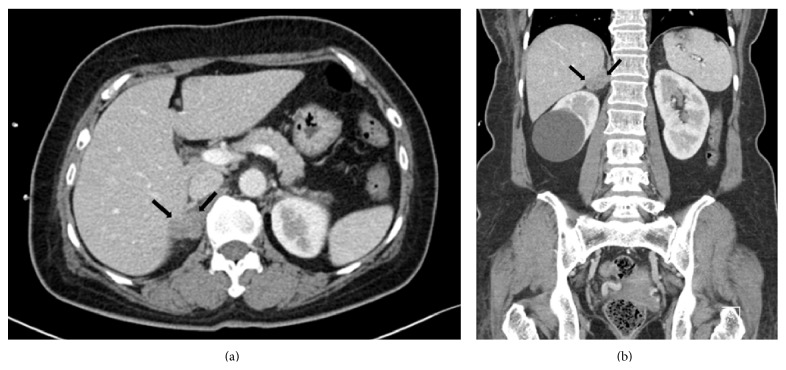
Axial (a) and coronal (b) abdominal computed tomography images during the late arterial phase show a 1.5 cm mass with a lobulated contour and mild enhancement in the right adrenal gland (black arrow).

**Figure 5:**
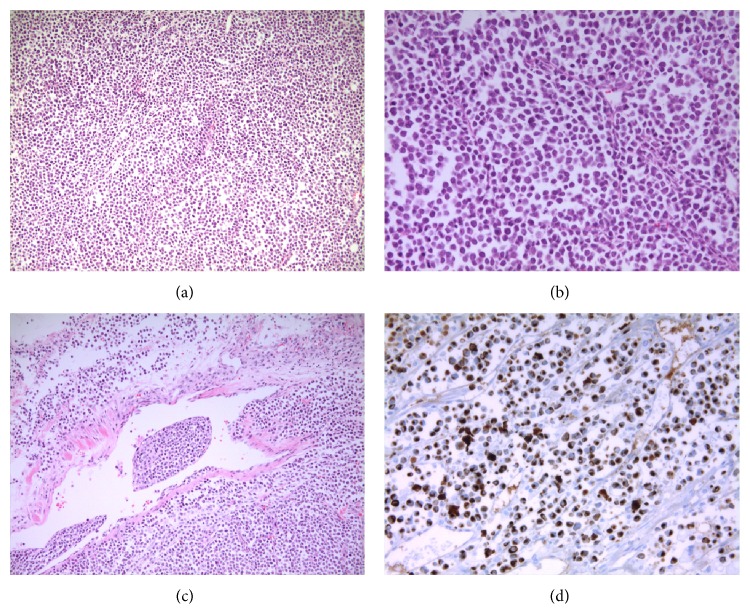
Microscopic findings of the right axillary mass showed diffuse sheets of basophilic tumor cells ((a) HE ×200) with large and pale staining nucleus and tiny nucleoli ((b) HE ×400). Lymphovascular invasion is an almost constant histological finding ((c) HE ×400). Characteristic paranuclear dot-like staining of CK20 is specific for MCC ((d) CK20 ×400).

**Figure 6:**
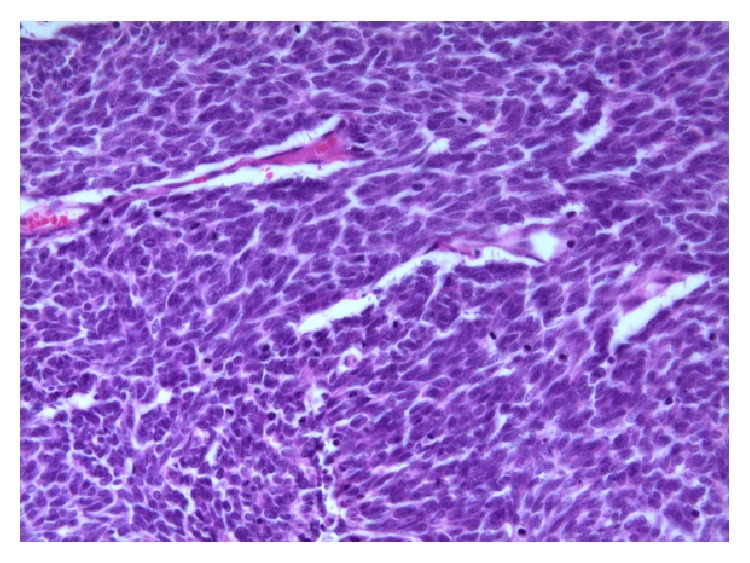
Histopathology of the adrenal gland mass showed similar findings observed in the axillary mass but had adopted more spindled cell morphology (HE ×400).

A 53-year-old woman presented with a palpable mass in the right axilla for 1 month. She did not have any particular history apart from hypertension, for which she had been taking antihypertensive medication once a day. On physical examination, a nontender right axillary mass of 10 cm was palpated. A slightly elevated level of carcinoembriogenic antigen (CEA) was detected (5.2 ng/mL; normal value is < 5.0 ng/mL), but no other abnormalities were detected in the laboratory studies. Mammography was performed, and a high-density mass was partially detected in the right axilla. Ultrasonography (US) showed a mass with an irregular shape, indistinct margin, internal hypoechogenicity, and increased peripheral vascularity (Figure 1). US-guided core needle biopsy was performed and pathologic examination indicated the presence of invasive carcinoma. Computed tomography (CT) scan of the chest was performed to characterize the right axillary mass and it indicated the presence of a 12 cm mass with a lobulated contour and heterogeneous enhancement in the right axilla (Figure 2). Positron emission tomography with the glucose analog 2-[fluorine-18] fluoro-2-deoxy-D-glucose (^18^F-FDG-PET) for preoperative staging showed a focal FDG-avid uptake in the right axilla with a maximum standardized uptake value (SUV max) of 12.7 and also showed a focal FDG-avid uptake in the right adrenal gland with an SUV max of 4.7 (Figure 3). For further evaluation of the newly found mass in the adrenal gland, CT of the abdomen and pelvis was performed and a 1.5 cm mass with lobulated contours and mild enhancement was observed in the right adrenal gland, directly invading the liver (Figure 4). The patient underwent a wide local excision of the right axillary lesion and a right adrenalectomy with liver resection and cholecystectomy. Histologic examination confirmed that the right axillary mass was a neuroendocrine carcinoma, composed of diffuse sheets of basophilic tumor cells with vesicular nuclei, small nucleoli, and scanty cytoplasm (Figures 5(a) and 5(b)). Lymphatic and vascular infiltrations were frequently identified (Figure 5(c)). Immunohistochemical staining of cytokeratin (CK) 20 was expressed in the paranuclear globules of the tumor cells in the punctate perinuclear dot-like pattern (Figure 5(d)). Tumor cells also showed diffuse expression of neuron specific enolase (NSE) but were negative for thyroid transcription factor-1 (TTF-1), CEA, and CK 7. Histologic examination of the right adrenal gland revealed tumor cells almost identical to the axillary mass except the slight spindle cell morphology (Figure 6). Paranuclear dot-like immunoreactivity of CK 20 and diffuse expression of NSE proved that they were compatible with separate two masses with the same origin. Metastasis from small cell carcinoma of lung could have been ruled out with the lack of expression of markers including TTF-1, synaptophysin, and chromogranin. Based on the histopathological and immunostaining findings, the two masses were diagnosed as MCC. Although the primary site of the tumor could not be clearly determined by histological examination, it is possible that the mass in the right axilla harbored a primary tumor that involved the dermis and subcutaneous fat. Nevertheless, the patient had no detectable primary skin lesion. After surgery, she received adjuvant radiation therapy.

Seven months after the surgery, a follow-up ^18^F-FDG-PET/CT was performed, which showed multiple FDG-avid lymph node uptakes in the left external iliac chain, right aortocaval area, left axilla, left supraclavicular area, bilateral jugular chain, right retropharyngeal space, and right palatine tonsil. An endoscopic biopsy was performed on the right palatine tonsil and it was diagnosed as metastatic MCC in pathologic report. The patient subsequently underwent palliative radiation therapy.

## 3. Discussion

Merkel cell carcinoma (MCC) is a rare and aggressive neuroendocrine carcinoma which was first described in 1972 by Toker as “trabecular carcinoma” [9]. Although the etiology and the mechanisms responsible for the regulation of its growth are currently unknown, exposure to sunlight and ultraviolet light, previous irradiation, infection with Merkel cell polyomavirus (MCV), and immunosuppression are likely to be significant risk factors [1, 6]. Although the two masses were confirmed as MCC by surgery, we did not further perform an anti-MCV assay in our patient. MCC usually affects elderly patients, with a mean age of 70 years at the time of diagnosis, as well as immunosuppressed individuals such as organ transplant recipients and HIV infected people [1, 10, 11]. It presents as a solitary, painless, pink to bluish papule or plaque on a sun-exposed area of the skin and grows rapidly [12]. Approximately 47% of these tumors occur in the head and neck, followed by the extremities (40%) and trunk (8%) [2]. Among the unusual extracutaneous primary sites, the parotid gland is the most common, followed by the submandibular gland, nasal cavity, mucosa of the lip, lymph nodes, and vulva/vagina [11]. In 14% of cases, the primary site is unknown and MCC presents at visceral or nodal sites [13]. Although MCC with an unknown primary site is unusual, no primary lesion can be identified in our patient after a thorough work-up. It is possible that the larger axillary mass was either a primary tumor involving the dermis and the subcutaneous fat or a metastatic MCC from an occult or regressed primary carcinoma [14]. Zhao and Meng reported the case of a MCC presenting as multiple lymph node metastases without a primary site [15].

To our knowledge, there have been a few cases of MCC involving the adrenal gland or the axilla [16, 17] but this is the first reported case that involves both axilla and adrenal gland. Moreover, detection and analysis of MCC through imaging has not been widely reported.

Although diagnostic imaging tool may be useful for staging, surgical planning, and proper management, there is no established imaging algorithm for MCC. Ultrasonography (US) is helpful for the evaluation of soft tissue that shows single or multicentric hypoechoic solid nodules arising from the dermis and extending into the subcutaneous fat layer [4–6]. Because of the cost effectiveness and possibility of real-time imaging during the procedure, some investigators prefer to use US in the initial staging work-up of MCC, especially in the head and neck region [18]. Moreover, contrast-enhanced computed tomography (CT) is useful for the evaluation of lymph nodes of the head and neck, nodular metastases in subcutaneous fat layers, and visceral metastases. On CT scans, the primary skin lesion presents as isodense or slightly hyperdense round nodules [4, 7]. High-attenuation lymphadenopathy is mostly detected in the axilla, followed by the neck (especially the parotid region), mediastinum, retroperitoneum, and groin [4, 9]. The presence of stranding of cutaneous fat adjacent to the primary site of the MCC on CT scans may suggest the presence of edema from lymphatic invasion [5]. Furthermore, imaging work-up of soft tissue lesions is best performed with magnetic resonance imaging (MRI). However, there are only a few studies describing the MRI findings in such cases, which have reported the presence of inhomogeneous signal intensities on T1 and T2 weighted images [8]. ^18^F-FDG-PET has an important role in diagnostic imaging of MCC, and ^18^F-FDG-PET/CT may also provide more accurate anatomic localization of tumors [4, 6]. Peloschek et al. reported that ^18^F-FDG-PET has a sensitivity of 85.7% and a specificity of 96.2% compared with those of 95.5% and 89.1% for conventional imaging modalities, respectively [18]. Based on the above mentioned findings, ^18^F-FDG-PET, US, CT, or MRI may be useful in patients with suspected metastatic MCC.

Although the treatment guidelines have not yet been defined, complete surgical excision is the best treatment option of MCC with a safety margin of 2–5 cm [19]. Histologically, the tumor cells are characterized by a large, pale nucleus with a scanty cytoplasm [10]. Mitotic activity is often marked and lymphatic and vascular invasions are common and this is an important prognostic indicator [20]. Immunohistochemistry has indicated that MCC expresses both epithelial (cytokeratins and epithelial membrane antigen) and neuroendocrine (neuron specific enolase, chromogranin, and synaptophysin) markers. CK 20 is a sensitive and specific marker for MCC and is helpful in distinguishing between MCC and other malignant and benign neoplasms [2]. Staining for leukocyte common antigen (LCA), TTF-1, and vimentin usually yields negative results [10]. Although the differential diagnosis includes other neuroendocrine tumors, such as small cell lung cancer, melanoma, cutaneous lymphoma, Ewing sarcoma, neuroblastoma, rhabdomyosarcoma, and basal cell carcinoma, the two masses were diagnosed as MCC, based on the histopathology and immunohistochemistry, as mentioned earlier [15, 21].

Previous studies demonstrated that male sex, tumor sizes larger than 2 cm, lymph node involvements, small cell pathology, lymphovascular invasions, and high mitotic rates are poor prognostic factors for MCC [2, 20]. Most cases of recurrent disease appeared within the first 6 to 12 months after initial diagnosis [22]. Therefore, short-term follow-up is recommended.

In summary, MCC is a rare, aggressive skin tumor with a high rate of metastasis and mortality. In the present paper, we describe a rare case of MCC of the axilla and adrenal gland and report the findings of US, CT, and ^18^F-FDG-PET/CT. Although MCC is usually diagnosed clinically and imaging studies of MCC have not been widely reported, a better understanding of MCC involvement in cutaneous and extracutaneous sites may be helpful for diagnosis and proper management.
